# Repurposed pharmacological agents for the potential treatment of COVID-19: a literature review

**DOI:** 10.1186/s12931-021-01885-8

**Published:** 2021-11-27

**Authors:** Alina Kröker, Madara Tirzīte

**Affiliations:** 1 Riga Stradins University, Riga, Latvia; 2 Riga East University Hospital, Clinical Centre “Gailezers”, Riga, Latvia

**Keywords:** SARS-CoV-2, COVID-19, Chloroquine, Hydroxychloroquine, Azithromycin, Lopinavir/ritonavir, Remdesivir, Tocilizumab, Corticosteroids, Anticoagulation

## Abstract

**Background:**

The COVID-19 pandemic has affected the world extraordinarily. This disease has a potential to cause a significantly severe course of disease leading to respiratory complications, multiple organ failure and possibly death. In the fight against this pandemic-causing disease, medical professionals around the world are searching for pharmacological agents that could treat and prevent disease progression and mortality. To speed the search of promising treatment options, already existing pharmacological agents are repurposed for the potential treatment of COVID-19 and tested in clinical trials. The aim of this literature review is to investigate the efficacy and safety of repurposed pharmacological agents for the treatment of COVID-19 at different pathophysiologic stages of the disease. For this literature review, online-databases PubMed and Google Scholar were utilised. Keywords “COVID-19”, “SARS-CoV-2”, “pathogenesis”, “drug targets”, “pharmacological treatment”, “cytokine storm”, “coagulopathy” and individual drug names were used. Scientific articles, including reviews, clinical trials, and observational cohorts, were collected and analysed. Furthermore, these articles were examined for references to find more clinical trials testing for the potential treatment of COVID-19. In total, 97 references were used to conduct this research paper.

**Results:**

The most beneficial pharmacological agent for the treatment of COVID-19 are corticosteroids, especially dexamethasone, for the treatment of mechanically ventilated COVID-19 patients. Other promising agents are remdesivir for the treatment of patients with COVID-19 pneumonia requiring minimal supplemental oxygen therapy, and IL-6 receptor antagonist monoclonal antibodies in severe COVID-19. Lopinavir/ritonavir, as well as chloroquine or hydroxychloroquine with or without azithromycin demonstrate the least efficacy in the treatment of COVID-19. The clinical benefits of the treatment of a COVID-19-specific coagulopathy with increased dosing of anticoagulation need further research and confirmation of randomised controlled trials.

**Conclusion:**

The search for pharmacological treatment of COVID-19 has elicited great controversy. Whereas drugs like chloroquine, hydroxychloroquine, and lopinavir/ritonavir have not shown proven benefit, the agents remdesivir and dexamethasone are recommended for clinical use for the treatment of COVID-19. Further randomised trials for other pharmacological treatment strategies are awaited.

## Background

The new strain of coronavirus, termed severe acute respiratory syndrome coronavirus two (SARS-CoV-2), has caused numerous cases of a respiratory illness, named Coronavirus disease 2019 (COVID-19), leading to a rapid spread of the disease [1]. On the 11th of March 2020, the World Health Organization (WHO) declared COVID-19 a pandemic [2]. Since then, COVID-19 has changed the world immensely and has taken a toll on healthcare systems. As of the 11th of March 2021, globally there has been over 117 million confirmed cases of COVID-19 of which over 2.6 million cases have resulted death [3]. COVID-19 has affected some countries worse than others leading to higher case fatalities when hospitals and intensive care units (ICU) could not carry the burden of this disease and shortages of medical equipment and ventilation systems became inevitable. As this disease has already claimed a vast number of lives, it has become an urgent task to evaluate potential pharmacological agents for the treatment of COVID-19 to improve clinical outcomes, decrease case fatalities, and contain the spread of the disease.

To speed the search of promising treatment and decrease the devastating effect of this potentially deadly disease, many clinical trials around the world are engaged in repurposing already existing drugs that had been developed for other indications and test them for the efficacy and safety in COVID-19.

The aim of this literature review is to investigate the efficacy and safety of repurposed pharmacological agents for the treatment of COVID-19 at different pathophysiologic stages of the disease.

For this literature review, online-databases PubMed and Google Scholar were utilised. Keywords “COVID-19”, “SARS-CoV-2”, “pathogenesis”, “drug targets”, “pharmacological treatment”, “cytokine storm”, “coagulopathy” and individual drug names were used. Scientific articles, including reviews, clinical trials, and observational cohorts, were collected and analysed. Furthermore, these articles were examined for references to find more clinical trials testing for the potential treatment of COVID-19. In total, 97 references were used to conduct this research paper.

This literature review focuses on pharmacological agents that have promising features for the treatment of COVID-19. These agents are organised according to their mechanism of action and hypothesised activity against SARS-CoV-2 and its sequelae. The first chapter is dedicated to the characteristics of SARS-CoV-2 and its pathogenic actions to illuminate possible important drug targets. Thereafter, drugs with hypothetical antiviral properties, including chloroquine and hydroxychloroquine with and without azithromycin, lopinavir/ritonavir, and remdesivir, drugs with immunomodulatory properties, tocilizumab and corticosteroids, as well as anticoagulants are analysed for the potential use of clinical treatment of COVID-19.

## SARS-COV-2

SARS-CoV-2, which was initially called 2019-nCoV, is the third coronavirus causing severe respiratory infections in humans in the past 20 years after severe acute respiratory syndrome coronavirus (SARS-CoV), leading to the SARS epidemic from 2003, and Middle East respiratory syndrome coronavirus (MERS-CoV), leading to the MERS epidemic from 2012. All three of these viruses belong to the genus of *Betacoronavirus* [4]. SARS-CoV-2, as all the members of the *Coronaviridae* family, is an enveloped positive sense, single-stranded ribonucleic acid (RNA) virus that shares a genome similarity of 79% with SARS-CoV and 50% with MERS-CoV [5]. Further genome sequencing demonstrated a similarity of 96.2% with a bat coronavirus, BatCoV RaTG13, indicating that bats may be the original hosts from which SARS-CoV-2 has emerged [6]. SARS-CoV and MERS-CoV both needed mammalian intermediate hosts to be able to transmit to humans. Since the close relative of SARS-CoV-2 found in bats has significant differences in its receptor-binding domain compared to the pandemic-causing virus, it is thought that SARS-CoV-2 may as well have developed necessary mutations within intermediate hosts to be able to infect humans [7–9]. Another theory suggests a period of cryptic transmissions in which the virus gradually mutated within humans and eventually acquired the functions to cause a significant disease [8, 9]. The exact mechanism how SARS-CoV-2 was able to affect humans is still unknown, but further research may become important to prevent future dangerous zoonotic transmissions of infections.

### Structure and life cycle

SARS-CoV-2 is characterised by the following structural proteins: spike (S), envelope (E), membrane (M), and nucleocapsid (N) proteins [10]. Angiotensin-converting enzyme two (ACE2) has been confirmed to be the receptor that facilitates SARS-CoV-2 entry into the host cells [6, 11]. ACE2 is specifically expressed on the surface of lung alveolar and small intestine epithelial cells, but also on vascular endothelial cells, smooth muscle cells, oral and nasal mucosa, and in kidneys and heart [12]. SARS-CoV-2 is thought to enter its target cell via endocytosis and via membrane fusion. The S protein of SARS-CoV-2 consist of subunits, S1 and S2. The S1 subunit contains the receptor-binding domain which binds to ACE2 for entry. The S2 subunit facilitates the fusion of the viral envelope with the host cell’s membrane. For the activation of these subunits, priming of the S protein by host cell surface proteases must occur. Transmembrane protease serine subtype two (TMPRSS2) cleaves the S protein at its S1/S2 and S2’ cleavage sites [13]. After this, the process of host cell entry can begin. The viral positive sense RNA is being uncoated and released into the host cell cytoplasm. Translation using host ribosomes proceeds and polyproteins are encoded [10]. The polyproteins are proteolysed to 16 non-structural proteins that are involved in replication and transcription, including viral proteases 3-chymotrypsin-like protease (3CLpro), also called main protease, and papain-like protease (PLpro), and RNA-dependent RNA polymerase (RdRp) [14]. RdRp replicates the positive sense to a negative sense RNA. This can be used in two ways. First, it can be used for the replication for more genomic positive sense RNA that will be facilitated in new virions. Second, it can be transcribed to subgenomic messenger RNAs via discontinuous transcription that encode for all viral structural proteins, S, E, M, and N. The structural proteins are encoded at the rough endoplasmic reticulum and transported further to the Golgi apparatus where assembly of the positive sense RNA and structural proteins continues to produce new virions. Finally, mature virions are transported within Golgi vesicles and released out of the host cell via exocytosis [14, 15].

### Pathogenesis and clinical course

COVID-19 most commonly presents at onset with fever, cough, myalgia, fatigue, and headache. As the disease progresses, dyspnoea and lymphopenia become prominent features in susceptible individuals that develop viral pneumonia [16–18]. Typical computed tomography (CT) findings in these with viral pneumonia are bilateral, mostly peripheral ground glass opacities, commonly mixed with consolidations, primarily in the lower lobes [19]. Complications can be severe, leading to multiple organ failure and death, and include acute respiratory distress syndrome (ARDS), acute cardiac injury, thromboembolic disturbances, secondary infections, shock, and inflammatory complications, such as cytokine release syndrome (CRS) or, also called, cytokine storm [16, 20]. CRS is an excessive uncontrolled cascade of pro-inflammatory cytokine and chemokine release, including the release of interleukin 6 (IL-6) [21].

Severe COVID-19 patients have higher risk of thrombosis. A reason for that is the disrupted alveolar-endothelial barrier leading to endothelial dysfunction and promotion of microcirculatory microthrombi [22]. ACE2 is also well expressed on endothelial cells [12]. When SARS-CoV-2 infects endothelial cells, the process will promote further endothelial damage and inflammation advocating a coagulation cascade. Thromboembolic complications are also aggravated by the cytokine storm, systemic inflammatory response syndrome (SIRS), and possible immobilisation and comorbidities of the patients [23].

### Potential drug targets

The pathogenetic processes occurring in the COVID-19 infection give rise to several potential drug targets. The first process that can be targeted is the viral entry and endocytosis of SARS-CoV-2 into the host cells. Chloroquine and hydroxychloroquine are drugs that are hypothesised to act against SARS-CoV-2 by inhibiting virus-host fusion [24]. Viral proteases are also important drug targets. SARS-CoV-2 encodes viral proteases 3CLpro and PLpro during its life cycle which are important for replication. Lopinavir/ritonavir is a drug combination primarily used against HIV which acts as a viral protease inhibitor. By inhibiting 3CLpro and PLpro, lopinavir/ritonavir could possibly stop viral replication [14, 24]. Essential for viral replication of SARS-CoV-2 is also the RdRp. Inhibiting this non-structural protein will bring the replication process of this virus to a standstill. A promising RNA polymerase inhibitor in the fight against COVID-19 is remdesivir [14, 25].

The cytokine storm is also an important pathogenic process of COVID-19 with devastating sequelae. Therefore, pharmacological agents with immunomodulatory effects are important to consider in the treatment of COVID-19. Promising immunomodulatory therapy strategies for COVID-19 include specific immunosuppression by the inhibition of IL-6 signal transduction with the use of tocilizumab and non-specific immunosuppressive strategies including corticosteroids [26].

## Pharmacological agents with antiviral properties

### Chloroquine and hydroxychloroquine with or without azithromycin

Chloroquine and its derivative, hydroxychloroquine, are 4-aminoquinolines that have been widely used in the prophylaxis and treatment of malaria. Besides their antimalarial activity, they also exert anti-inflammatory action. Thus, hydroxychloroquine is also frequently used in the treatment of rheumatic diseases, such as rheumatoid arthritis and systemic lupus erythematosus [27]. The broad antiviral properties of chloroquine and its derivative have also been recognised. In the fight against SARS-CoV-2, they are thought to inhibit viral entry and release from host cells by increasing the pH in endosomes, lysosomes, and Golgi vesicles. Endocytosis is also thought to be prevented by the inhibition of ACE2 glycosylation. Furthermore, they have promising anti-inflammatory effects that could potentially combat a cytokine storm by acting against the release of inflammatory markers [14].

Chloroquine had already shown potential in the treatment and prophylaxis of SARS, as it successfully inhibited in vitro infection and spread of SARS-CoV in two separate studies [28, 29]. Chloroquine has also demonstrated to be effective against SARS-CoV-2 in vitro. A study conducted by Wang and colleagues proved that chloroquine could inhibit SARS-CoV-2 infection at entry and post-entry stages in Vero E6 cells and advocated the run of clinical trials for chloroquine against COVID-19 [30]. Yao and colleagues confirmed identical findings by testing chloroquine, but also hydroxychloroquine, in vitro in Vero cells against SARS-CoV-2. Furthermore, they implicated that hydroxychloroquine had even more potency in comparison to chloroquine in in vitro activity against the COVID-19-causing virus [31]. Liu and colleagues as well performed in vitro analyses of chloroquine and hydroxychloroquine in African green monkey kidney VeroE6 cells in relation to SARS-CoV-2 infection. They concluded that hydroxychloroquine, being less toxic than chloroquine, could competently inhibit infection but they warned for extensive use and dosing, as this could lead to dangerous poisoning [32].

A Brazilian double-blinded, phase IIb, randomised clinical trial tested for the efficacy and safety of chloroquine diphosphate in severe SARS-CoV-2 infected patients with two different dosages. 81 participants were enrolled in this study, in which 41 received high-dose chloroquine diphosphate 600 mg twice a day for 10 days and 40 received low-dose chloroquine diphosphate 450 mg twice a day on the first day and once a day for 4 days. 100% of the participants received azithromycin, whereas 86.8% in the low-dose and 92.5% in the high-dose group also received oseltamivir simultaneously to exclude other infections. The study concluded higher lethality in the high-dose group with 39% compared to the low-dose group with 15%. Therefore, high dose treatment in severe COVID-19 patients was discontinued as the risks were higher than the benefits. These findings could have been influenced by the concurrent administration of azithromycin and oseltamivir. Also, the randomisation of this study lead to the assignment of more of older patients with cardiac comorbidities to the high-dose group which could have also affected the results. On the other hand, typical severe COVID-19 patients are individuals with higher age and comorbidities, thus, the study excluded the general use of high-dose chloroquine for severe COVID-19 patients. Furthermore, in neither group viral clearance by day four was attained. This study did not conclude any benefits of chloroquine diphosphate in severe COVID-19 patients, but they suggested further testing of chloroquine as a prophylactic drug and its use in mild to moderate COVID-19 patients [33] (Table 1).

**Table 1:** Summary of clinical studies: chloroquine and hydroxychloroquine with or without azithromycin

Study	Study design (number of participants)	Study arms (number of participants)	Endpoints	Results
Borba et al. [33]	Randomized phase IIb, double-blinded, clinical trial (n = 81)	1. High-dose chloroquine diphosphate 600 mg twice daily for 10 days (n = 40) 2. Low-dose chloroquine diphosphate 450 mg twice daily on day 1 and once daily for 4 days (n = 41)	Primary: Lethality by day 28 Secondary: Lethality on day 13; participant clinical status; laboratory examinations; ECG on days 13 and 28; daily clinical status during hospitalization; duration of mechanical ventilation and supplementary oxygen (if applicable); time in days from treatment initiation to death	Higher lethality in high-dose group (39%) compared to low-dose group (15%) More QTc interval prolongation in high-dose group than in low-dose group (18.9% vs 11.1%)
Huang et al. [34]	Randomized controlled trial (n = 22)	1. Chloroquine 500 mg orally twice daily for 10 days (n = 10) 2. Lopinavir/Ritonavir 400/100 mg orally twice daily for 10 days (n = 12)	Viral clearance; time of hospital discharge; clinical recovery; lung clearance on CT	Superiority of chloroquine in viral clearance by day 14 (100% vs 91.7%), lung improvement on CT by day 14 (100% vs 75%), and earlier hospital discharge at day 14 (100% vs 50%)
Tang et al. [36]	Randomised, open-label, controlled trial (n = 150)	1. Standard of care (n = 75) 2. Standard of care plus hydroxychloroquine 1200 mg daily for 3 days, then 800 mg daily for 2 to 3 weeks (n = 75)	Primary: Negative conversion of SARS-CoV-2 by day 28 Secondary: Probability of negative conversion at day 4, 7, 10, 14, or 21; probabilities of alleviation of clinical symptoms; improvement of C reactive protein, erythrocyte sedimentation rate, tumour necrosis factor α, interleukin 6, and absolute blood lymphocyte count; improvement of lung lesions on chest radiology; all cause death	Similar results between the study arms 28-day negative conversion rate was 85.4% (73.8–93.8%) in hydroxychloroquine vs 81.3% (71.2–89.6%) in the standard of care group Negative conversion rates at specific days were similar between the groups Alleviation of symptoms at 28 days was 59.9% (45–75.3%) with hydroxychloroquine vs 66.6% (39.5–90.9%) with standard of care More adverse events in the hydroxychloroquine than in the standard of care arm (30% vs 9%)
Geleris et al. [37]	Observational cohort study (n = 1376)	1. Hydroxychloroquine 600 mg twice on day one, then 400 mg daily for 4 days (n = 811) 2. No hydroxychloroquine (n = 565)	Time from study baseline to intubation or death	No significant differences between the groups for the risk of intubation or death. In total, 346 patients (25.1%) developed respiratory failure
Gautret et al. [38]	Non-randomised, open-label, clinical trial (n = 36)	1. Hydroxychloroquine sulphate 200 mg 3 times a day for 10 days (n = 20); six participants additionally received azithromycin 500 mg on the first day, then 250 mg per day for 4 days 2. Control group (n = 16)	Primary: Virological clearance at day six post-inclusion Secondary: Virological clearance over time; clinical follow-up; occurrence of side effects	70% of hydroxychloroquine group had virological clearance at day 6 (100% in the group with azithromycin and 57.1% in the hydroxychloroquine-only group) compared to 12.5% in the control group
Molina et al. [41]	Prospective observational study (n = 11)	Hydroxychloroquine 600 mg per day for 10 days and azithromycin 500 mg on day one and 250 mg on days 2 to five	Virologic and clinical outcomes	No patient had virological clearance after 6 days
Cavalcanti et al. [42]	Randomised, open-label, controlled trial (n = 665)	1. Standard of care (control group) (n = 227) 2. Standard of care plus hydroxychloroquine 400 mg twice daily for 7 days (n = 221) 3. Standard of care plus hydroxychloroquine (dosage as second group) plus azithromycin 500 mg once a day for 7 days (n = 217)	Primary: Clinical status at day 15 Secondary: Clinical status at day 7; intubation within 15 days; use of supplemental oxygen or non-invasive ventilation within 15 days; use of mechanical ventilation within 15 days; duration of hospital stay; in-hospital death; thromboembolic complications; acute kidney injury; number of days alive and free from respiratory support up to 15 days	No significant difference between the groups regarding clinical improvement at day 15, nor in any of the secondary outcomes Increased frequency of QTc prolongation and increased liver enzymes in the second and third group
Boulware et al. [43]	Randomized, double-blind, placebo-controlled trial (n = 821)	1. Hydroxychloroquine loading dose of 800 mg with subsequent 600 mg after 6 to 8 h, then 600 mg once a day for 4 days (n = 414) 2. Placebo (n = 407)	Primary: Symptomatic illness confirmed by PCR or COVID-19-related symptoms Secondary: Incidence of hospitalization for COVID-19 or death; incidence of PCR-confirmed SARS-CoV-2 infection; incidence of COVID-19 symptoms; the incidence of discontinuation of the trial intervention due to any cause; severity of symptoms, if applicable, at days 5 and 14	No significant difference in incidence of new illness compatible with COVID-19 between hydroxychloroquine and placebo (11.8% vs 14.3%) as post-exposure prophylaxis
Chorin et al. [44]	Retrospective cohort study (n = 251)	Hydroxychloroquine 400 mg twice a day on day 1, followed by 200 mg twice daily for 4 days, plus azithromycin 500 mg once a day for 5 days	Extreme QTc interval prolongation on ECG	Significant correlation between drug treatment and prolongations of QTc. Extreme QTc prolongation in 23% of patients

Another randomised study conducted in China tested for the efficacy and safety of chloroquine in COVID-19 patients in comparison to the use of lopinavir/ritonavir. They reported that chloroquine was superior in viral clearance with 100% clearance by day 13 versus 91.7% by day 14 of the control group, lung improvement on CT with 100% clearance in comparison to 75% of the control group by day 14, and earlier hospital discharge with 100% versus only 50% of the lopinavir/ritonavir group at day 14. This study was heavily limited by the small sample size of only 22 participants. 10 participants, three of which were in severe and seven in moderate condition, were in the chloroquine and 12, five of which with severe and seven with moderate disease, in the lopinavir/ritonavir group [34] (Table 1). Similar results of chloroquine being potent against COVID-19 were reported as studies including more than 100 patients in more than 10 hospitals throughout China concluded superiority against control treatment. This led to the recommendation of chloroquine for the treatment of pneumonia caused by COVID-19 for larger populations [35]. The details of the studies were not mentioned which may raise questions to the accuracy of this proposition.

The efficacy and safety of hydroxychloroquine against COVID-19 was analysed in a randomised controlled trial organised in three Chinese provinces. Of 150 participants hospitalised for COVID-19, 75 received standard care of intravenous fluids, supplemental oxygen, regular laboratory, and SARS-CoV-2 testing, monitoring of haemodynamic stability, and intensive care, whereas 75 participants received standard care in addition to hydroxychloroquine, 1200 mg daily for 3 days, then 800 mg daily for a total of 2 weeks, or 3 weeks for severe patients. Most of the participants had mild to moderate symptoms and two had severe COVID-19. The mean time between onset of symptoms and randomisation was 16.6 days. This study concluded no significant differences in negative viral conversion of the hydroxychloroquine group compared to the control group. Two patients in total, both from the chloroquine group, experienced serious side events with disease progression and upper respiratory tract infection. More non-serious side events, such as gastrointestinal symptoms, were also experienced in the hydroxychloroquine group [36] (Table 1). This study is limited as it is not double-blinded and because the time between disease onset to randomisation is extensively long. Nevertheless, a treatment addition of hydroxychloroquine to standard care of persistent mild to moderate COVID-19 infection was not recommended.

An observational cohort study operated in New York City concentrated on the correlation of the use of hydroxychloroquine and intubation or death in SARS-CoV-2 positive, hospitalised patients. In this analysis, 1376 patients were included. 811 of these received hydroxychloroquine 600 mg on the first day, followed by 400 mg daily for 4 days. 565 patients did not receive hydroxychloroquine. Treatment started for 45.8% of the hydroxychloroquine group within 24 h and for 85.9% within 48 h from the presentation to the emergency department. The patients were observed for a median time of 22.5 days. This study did not conclude any significant differences between the groups for the risk of intubation or death [37] (Table 1).

In France, Gautret and colleagues investigated the effect of hydroxychloroquine on the nasopharyngeal viral load of COVID-19 hospitalised patients in an open-label, non-randomised clinical trial. Out of 36 evaluated participants, 20 were treated with oral hydroxychloroquine sulphate 200 mg three times a day for 10 days, six of which additionally received azithromycin 500 mg on the first day, then 250 mg per day for 4 days. The remaining 16 patients were in the control group. After 6 days, of the patients treated with hydroxychloroquine and azithromycin 100% were virologically negative in comparison to 57.1% of the hydroxychloroquine and 12.5% of the control group. They concluded that hydroxychloroquine was efficient in reducing viral load of SARS-CoV-2 with azithromycin enhancing this result and recommended the use of this combination for COVID-19 treatment and reduction of transmission [38] (Table 1). However, this study has been criticised to be methodologically flawed and “non-informative” by reviewers [39, 40]. On the basis of the aforementioned results, Molina and colleagues conducted a prospective observational study in which they examined virologic and clinical outcomes in 11 hospitalised severe COVID-19 patients treated with hydroxychloroquine and azithromycin in the same dose regime. They contradicted the results of Gautret and colleagues as none of their patients had virological clearance after 6 days of treatment. Of these patients, one had died, two needed ICU transfer, and one had to discontinue the combination treatment because of QT interval prolongation on ECG. Therefore, they established that there is no strong evidence that supports hydroxychloroquine and azithromycin in combination for the treatment of severe COVID-19 patients [41] (Table 1).

Another study evaluated the efficacy and safety of hydroxychloroquine and azithromycin for COVID-19 treatment. In this multicentre, randomised, open-label, controlled trial, 665 hospitalised patients with confirmed and suspected mild to moderate COVID-19 were divided into three groups. The first group with 227 participants received standard care. The standard care was decided by the clinicians and the use of glucocorticoids, immunomodulators, antibiotics, and antivirals was permitted. The second group with 221 participants received standard care and hydroxychloroquine 400 mg two times a day for 7 days. The third group with 217 participants additionally received azithromycin 500 mg once a day for 7 days. 504 participants had confirmed COVID-19, 87.8% were enrolled in the study within 10 days, with a median time of 7 days of the onset of symptoms, and all participants received either no supplemental oxygen or maximally 4 l per minute. After 15 days of treatment, the clinical status was assessed using a seven-point ordinal scale. The study concluded no difference between the groups regarding the clinical improvement of the patients. In the second and third group, the frequency of corrected QT interval (QTc) prolongation and increased liver enzymes were higher [42] (Table 1).

If hydroxychloroquine had potential for the postexposure prophylaxis for COVID-19 was inspected in a double-blinded, randomised, placebo-controlled clinical trial in the United States and Canada. Recruitment of participants was conducted via online surveys to reach as many candidates as possible. 821 participants were enrolled in this study. All of them were asymptomatic and had either high-risk or moderate-risk exposure to COVID-19. Exposure was defined as household or occupational contact less than six feet to a COVID-19-infected individual for more than 10 min with neither face mask or eye shield, accounting for high-risk, or with face mask but no eye shield, being moderate-risk exposure. High-risk exposure candidates considered for 87.6% of all participants. Randomisation occurred 4 days after exposure. 414 participants received hydroxychloroquine in a loading dosage of 800 mg, with subsequent 600 mg after 6 to 8 h, followed by 600 mg once a day for 4 days. 407 participants received a placebo. The participants were contacted after 1, 5, 10, and 14 days to evaluate their health status with follow-up surveys. Due to lack of diagnostic tests, not all participants were able to get polymerase chain reaction (PCR) tests for SARS-CoV-2. After 14 days, 107 participants, counting 13.0% of all recruited, had PCR-confirmed or probable COVID-19 with symptoms compatible with the disease. Of these, 49, or 11.8%, of the participants were in the hydroxychloroquine group, and 58, or 14.3%, were in the placebo group. The difference between the groups in disease incidence was not significantly different. Also, the hydroxychloroquine group experienced more side effects by day 5 in comparison to the placebo group, the first accounting for 40.1% and the latter for 16.8%. These side effects included gastrointestinal disturbances, such as nausea, abdominal discomfort, and loose stools [43] (Table 1). However, this study has its limitations. Because of the internet-based fashion of this study, mostly younger, healthier individuals were recruited. That is why this study cannot exclude the efficacy of hydroxychloroquine for postexposure prophylaxis in more susceptible individuals, such as people with older age or with comorbidities. Also, due to lack of testing possible asymptomatic COVID-19 carriers could not be fully detected. On the other hand, most of the participants were health care workers. As these individuals are the ones with higher exposure risk to sick people, the search for possible COVID-19 prophylaxis to decrease healthcare burden, and the fact that this study did not conclude a significant benefit for this group by taking hydroxychloroquine to prevent COVID-19, is very important.

As a response to more and more clinical trials involved in the use of hydroxychloroquine for COVID-19, especially in combination with azithromycin, the concerns about serious side effects, including life-threatening rhythmic disturbances, are rising. A retrospective cohort study performed in hospitals in the United States and Italy focused on this concern. 251 COVID-19 patients which were hospitalised and treated with hydroxychloroquine and azithromycin were included. Dosages were orally 400 mg twice a day on the first day, followed by 200 mg twice a day for 4 days for hydroxychloroquine, and orally 500 mg of azithromycin once a day for 5 days. Mandatory for inclusion was also a baseline ECG and a minimum of one follow-up ECG of treatment. The results presented a significant correlation between the drug treatment and prolongations of QTc. 58 of 251 patients, accounting for 23%, experienced an extreme prolongation of QTc of more than 500 ms with previous normal QTc on baseline ECG. One patient in this group developed a polymorphic ventricular tachycardia, characteristic to torsade de point, and needed cardioversion. The measured prolongation also correlated partially retrogressively with treatment cessation. The study concluded that the combination of hydroxychloroquine and azithromycin for the treatment of COVID-19 expresses significant risk for the development of QTc prolongation, which in turn increases the risk for the development of life-threatening arrythmia and recommends a risk stratification before necessary administration [44] (Table 1).

As different clinical trials are presenting different results, the use of chloroquine or hydroxychloroquine with or without azithromycin in COVID-19 patients trigger a great controversy. In July 2020, after many clinical trials were already conducted, two research papers are presenting results that many clinicians already suspected. Although, chloroquine and hydroxychloroquine have presented to be effective in VeroE6 cells of African green monkey kidneys, these pharmacological agents have shown to not have in vitro effect on SARS-CoV-2-infected human airway epithelial cells [45, 46]. The researchers oppose that chloroquine and hydroxychloroquine have antiviral effect in the human respiratory system. This is explained by the low levels of cysteine protease cathepsin L in airway epithelial cells. This endosomal enzyme activates the S protein of SARS-CoV-2 for viral entry in a pH-dependent pathway. Contrarily, viral entry of SARS-CoV-2 in airway epithelial cells appear to occur in a pH-independent pathway via TMPRSS2 [45]. Thus, chloroquine and hydroxychloroquine cannot express their pH-modulating effects in inhibiting viral entry in human airway epithelial cells. Furthermore, testing of hydroxychloroquine with or without azithromycin in SARS-CoV-2-infected macaques presented that the drug did not affect levels of viral load nor had treatment efficacy in early or late infections, or as post-exposure prophylaxis [46].

From the update of the COVID-19 Treatment Guidelines of the National Institutes of Health (NIH) from October 2020, the use of chloroquine and hydroxychloroquine in hospitalised and non-hospitalised COVID-19 patients with or without azithromycin, as well as the use of high-dose chloroquine for the treatment of COVID-19 are not recommended. Exceptions are the use of chloroquine or hydroxychloroquine in non-hospitalised patients in clinical trials [47]. This opinion is supported by the European Respiratory Society (ERS) whose COVID-19 management guideline do not recommend hydroxychloroquine in hospitalised and non-hospitalised patients, azithromycin in hospitalised patients in the absence of bacterial infection, nor the combination of hydroxychloroquine and azithromycin in COVID-19 patients [48].

### Lopinavir/ritonavir

Lopinavir and ritonavir are antiretroviral agents that inhibit viral proteases. Lopinavir is used in combination with ritonavir to enhance its effect in the treatment for HIV [25]. Lopinavir/ritonavir had previously shown to have activity against SARS-CoV in vitro [49]. A consecutive retrospective matched cohort study concluded that lopinavir/ritonavir in addition to standard treatment had improved the clinical outcomes of SARS patients [50]. In the fight against SARS-CoV-2, this drug combination has demonstrated efficacy against its 3CLpro on molecular level [51]. These findings spark hope in the treatment of COVID-19 and led to in vivo studies of lopinavir/ritonavir against this disease.

An open-label, randomised, controlled trial in China, named LOTUS, assessed the efficacy and safety of lopinavir/ritonavir in SARS-CoV2-infected, hospitalised, seriously ill patients. Eligible participants were those with positive reverse transcription PCR (RT-PCR) test for SARS-CoV-2, imaging-confirmed pneumonia, and a peripheral capillary oxygen saturation (SpO2) of 94% or less. Of 199 enrolled participants, 99 were grouped to receive lopinavir/ritonavir 400 mg and 100 mg two times a day for 14 days, and 100 participants received standard care. Five patients of the lopinavir/ritonavir group did not receive doses due to early death and the refusal of physicians. In median time, participants were randomised after 13 days from the onset of symptoms. The participants were evaluated for clinical improvement, viral load, adverse events, and mortality. The median time to clinical improvement was 15 days in the lopinavir/ritonavir group and 16 days in the control group. Also, the mortality after 28 days and the nasopharyngeal viral load did not differ significantly between the groups with 19.2% in the lopinavir/ritonavir in comparison to 25% in the standard of care group. The lopinavir/ritonavir group had more commonly adverse events, being mostly gastrointestinal complaints. Serious adverse events, including ARDS, acute kidney injury, and secondary infections, were more common in the standard care group than in the treatment group with 32.3% versus 20% of occurrence, respectively. All in all, the study did not conclude a significant benefit of lopinavir/ritonavir in comparison to standard care in clinical improvement, mortality, and decreasing viral load in the treatment of seriously ill COVID-19 patients [52] (Table 2). However, since the median time for randomisation was relatively long for usual antiviral treatment and the participants had more severe disease, the study cannot exclude the efficacy of this drug combination in earlier administration and in milder diseased patients. Also, as the pandemic is having a vast impact on the hospital burden around the world, a hospital-stay shortened by 1 day could still have benefits [53].

**Table 2:** Summary of clinical studies: lopinavir/ritonavir

Study	Study design (number of participants)	Study arms (number of participants)	Endpoints	Results
Cao et al. LOTUS [52]	Randomised, open-label, controlled trial (n = 199)	1. Standard of care plus lopinavir/ritonavir 400 mg/100 mg twice a day for 14 days (n = 99) 2. Standard of care (n = 100)	Primary: Time to clinical improvement Secondary: Day 28 mortality; clinical improvement on day 7, 14, 28; ICU length of stay; duration of invasive mechanical ventilation; oxygen support in days; hospital stay in days; time from randomization to death	No significant difference in time to clinical improvement between lopinavir/ritonavir and standard of care (median time in days, 15 vs 16) 28-day mortality lower in lopinavir/ritonavir than in standard of care (19.2% vs 25%) Shorter ICU stays with lopinavir/ritonavir (median time in days, 6 vs 11) Shorter duration from randomisation to hospital discharge with lopinavir/ritonavir (median time in days, 12 vs 14) Higher percentage of patients with clinical improvement at day 14 with lopinavir/ritonavir (45.5% vs 30%) No significant differences for duration of oxygen therapy, duration of hospitalization, time from randomization to death
Yan et al. [54]	Retrospective cohort study (n = 120)	1. Lopinavir/ritonavir 400 mg/100 mg twice a day for a median duration of 10 days (n = 78) 2. Systemic corticosteroids (n = 54)	Time from symptom onset to SARS-CoV-2 negativity	The lack of lopinavir/ritonavir treatment was a risk factors for prolonged SARS-CoV-2 shedding
Li et al. [55]	Randomised, partially blinded, controlled trial (n = 86)	1. Lopinavir/ritonavir 200 mg/50 mg orally twice per day for 7 to 14 days (n = 34) 2. Arbidol 200 mg three times per day for 7 to 14 days (n = 35) 3. No antiviral treatment (n = 17)	Primary: Rate of positive to negative conversion of SARS-CoV-2 from initiation of treatment to day 21 Secondary: Rate of positive to negative conversion of SARS-CoV-2 at day 14; rate of antipyresis; rate of cough alleviation; improvement rate of chest CT at days 7 and 14; rate of deterioration of clinical status during time of study	No statistically significant difference in mean time for positive to negative conversion (9.0 days in lopinavir/ritonavir, 9.1 days in arbidol, 9.3 days in the control group) Positive to negative conversion after 14 days was 85.3% in lopinavir/ritonavir, 91.4% in arbidol, 76.5% in the control group No statistically significant difference in other secondary endpoints

120 SARS-CoV-2-positive, non-critically ill, hospitalised patients in Wuhan, China were analysed in a retrospective cohort study. A comparison of viral RNA shedding was undertaken between patients that received lopinavir/ritonavir and those who did not. Furthermore, clinical characteristics and general risk factors for prolonged viral shedding, which was defined as more than 23 days, was performed. 78 of these 120 patients received oral lopinavir/ritonavir 400 mg and 100 mg twice a day for a median duration of 10 days. Treatment initiation was performed within a median time of 10 days from the onset of symptoms. 54 of the 120 patients received systemic corticosteroids. In comparison to the control group, the lopinavir/ritonavir group had shorter time frames of viral shedding with a median duration of 22 days versus 28.5 days from the onset of symptoms. This shortening was only seen in patients who had initiated treatment within 10 days of symptom onset. Treatment initiation exceeding 10 days from onset of symptoms had no difference in the viral shedding median duration from the control group. Furthermore, older age of patients was also identified as a risk factor for prolonged viral shedding with a median age of 56 for prolonged shedding versus 48 for shedding of 23 days or shorter. The presence of comorbidities and the use of systemic corticosteroids were not associated with prolonged shedding. The study concludes that older age and the absence of lopinavir/ritonavir treatment were associated with prolonged viral shedding of SARS-CoV-2 [54] (Table 2).

The exploratory, randomised, partially blinded, controlled trial in China, named ELACOI, researched the efficacy and safety of lopinavir/ritonavir and, also, arbidol, as monotherapies in mild to moderate, hospitalised COVID-19 patients. The 86 enrolled participants were randomised into three groups. The first group included 34 participants and received lopinavir/ritonavir 200 mg and 50 mg orally twice per day for 7 to 14 days. The second group with 35 participants received arbidol, a hemagglutinin inhibitor previously known to have actions against influenza and SARS in vitro, in a dosage of 200 mg three times per day for 7 to 14 days. And the third group with 17 participants did not receive any antiviral medications. The different groups had all similar baseline characteristics with mild to moderate disease expression of COVID-19. Follow-up of this study was for 21 days. The mean time of conversion of SARS-CoV-2 positivity to negativity by pharyngeal RT-PCR was 9.0 days, 9.1 days, and 9.3 days for the lopinavir/ritonavir, arbidol, and control group, respectively, and did not have any statistically significant difference. Also, the negative conversion at day 7 and day 14 showed no statistically significant difference between the groups. Furthermore, there was no statistical difference in the groups regarding clinical improvement, such as rate of antipyresis, rate of cough resolution, and rate of chest CT improvement at day 7 and at day 14. Of the lopinavir/ritonavir group eight patients, accounting for 23.5%, deteriorated to severe or critical clinical status in comparison to three, 8.6%, and two, 11.8%, participants in the arbidol and control group, respectively. In this study, the treatment of lopinavir/ritonavir and arbidol as monotherapies did not shorten SARS-CoV-2 pharyngeal conversion, nor improve clinical status. More patients in the lopinavir/ritonavir group progressed to more severe disease and had more side effects, such as nausea, vomiting, abdominal pain, and diarrhoea [55] (Table 2). But this study did not elaborate the initiation of treatment from symptom onset, which could have affected the results of this study.

For the treatment of COVID-19, the NIH recommends against the use of lopinavir/ritonavir and other HIV protease inhibitors in hospitalised and non-hospitalised patients according to the guideline update from February 2021 [56]. The ERS supports this viewpoint and does not recommend lopinavir/ritonavir for hospitalised COVID-19 patients [48]. Furthermore, a pharmacokinetic investigation in which eight COVID-19 patients received lopinavir/ritonavir in a dosage of 400 mg/100 mg orally two times per day for 3 to 10 days, concluded that, although plasma concentration of the drug in these patients were higher in comparison to HIV patients, the drug combination in this dosage could not reach plasma concentrations enough to inhibit the replication of SARS-CoV-2 [57]. Also, in a recent scientific article, published in June 2020, researchers state that lopinavir and ritonavir had no effect on the inhibition of SARS-CoV-2’s main protease, or 3CLpro [58]. Therefore, lopinavir/ritonavir is not a good candidate for the treatment of COVID-19.

### Remdesivir

Remdesivir is an adenosine analogue that has action against a broad spectrum of viruses. It is specifically known from clinical trials for the treatment of Ebola. By competing with adenosine triphosphate, remdesivir inhibits RdRp and halts viral replication [59, 60]. This pharmacological agent has proven to have efficacy in vitro and in animal studies with nonhuman primates against MERS-CoV [61, 62]. Remdesivir has also shown to have activity against other previous coronaviruses, including human coronaviruses, such as SARS-CoV, and zoonotic “pre-pandemic” coronaviruses, including bat CoVs [63]. In vitro activity against SARS-CoV-2 at post-entry stages in Vero E6 cells has been stated [30]. Furthermore, antiviral efficacy of remdesivir in SARS-CoV-2-infected human nasal and bronchial airway epithelial cells has been proven [64]. These findings make remdesivir a promising agent in the fight against COVID-19.

In a multicentre study conducted in Hubei, China, a randomised, double-blinded, placebo-controlled trial was performed to detect the effectiveness and safety of remdesivir in severe, hospitalised COVID-19 patients. Patient with SARS-CoV-2 RT-PCR positivity, pneumonia on chest imaging, a SpO2 equal to or beneath 94%, and in which the time between symptom onset and randomisation was within 12 days were enrolled. 155 participants started treatment of intravenous remdesivir with a loading dose of 200 mg for the first day, followed by 100 mg for 9 days. The control group with 78 participants received the same volume of placebo infusion for 10 days. Assessment of clinical improvement within 28 days was undertaken. The improvement of clinical status was defined as the reduction of two points in a six-point ordinal scale or the discharge from the hospital. Between the groups there was no statistically significant difference in the time of clinical improvement. Thus, remdesivir was concluded to not have clinical benefits in this study. Adverse events occurred equally in both groups with 66% in the remdesivir and 64% in the placebo group, including hypoalbuminaemia, constipation, hypokalaemia, anaemia, and increased total bilirubin [65] (Table 3).

**Table 3:** Summary of clinical studies: remdesivir

Study	Study design (number of participants)	Study arms (number of participants)	Endpoints	Results
Wang et al. [65]	Randomised, double-blind, placebo-controlled (n = 237)	1. Remdesivir single daily infusions 200 mg on the first day, followed by 100 mg on days 2 to 10 (n = 158) 2. Placebo (n = 79)	Primary: Time to clinical improvement within 28 days Secondary: Proportions of patients in each category on a six-point ordinal scale at day 7, 14, and 28; all-cause mortality at day 28; frequency of invasive mechanical ventilation; duration of oxygen therapy; duration of hospital admission; proportion of patients with nosocomial infection	Similar results in clinical improvement (median, 21 days in remdesivir vs 23 days in the placebo group) Patients receiving remdesivir within 10 days of symptom onset had a faster time to clinical improvement than those receiving placebo, without statistical significance (median, 18 days vs 23 days) Similar 28-day mortality between remdesivir and placebo (14% vs 13%)
Beigel et al. ACTT-1 [66]	Randomized, double-blind, placebo-controlled trial (n = 1062)	1. Remdesivir 200 mg loading dose on the first day, followed by 100 mg daily for up to 9 additional days (n = 541) 2. Placebo (n = 521)	Primary: Time to recovery Secondary: Clinical status at day 15; time to improvement of one and of two categories from the baseline ordinal score; clinical status and mean change in status on the ordinal scale at days 3, 5, 8, 11, 15, 22, and 29; time to discharge; number of days up to day 29 with and incidence and new onset of supplemental oxygen, with non-invasive ventilation or high-flow oxygen, with invasive ventilation or ECMO; number of days of hospitalization up to day 29; mortality at days 14 and 28	Shorter time to recovery with remdesivir than with placebo (median, 10 days vs 15 days) Odds of improvement in the ordinal scale score were higher in the remdesivir than in the placebo group Mortality by day 15 were lower in the remdesivir group than in the placebo group (6.7% vs 11.9%) Shorter time to clinical improvement with remdesivir than placebo (one-category improvement: median, 7 vs 9 days; two-category improvement: median, 11 vs 14 days) Shorter time to hospital discharge with remdesivir (median, 8 days vs 12 days) Fewer days of oxygen supplementation with remdesivir from baseline (median, 13 days vs 21 days) Lower new incidences of oxygen supplementation in remdesivir group (36% vs 44%) Equal median duration of non-invasive ventilation or high-flow oxygen from baseline in both groups (6 days vs 6 days) Lower new incidences of non-invasive ventilation or high-flow oxygen use with remdesivir (17% vs 24%) Fewer days of mechanical ventilation or ECMO from baseline with remdesivir (median, 17 days vs 20 days) Lower new incidences of mechanical ventilation or ECMO with remdesivir than with placebo (13% vs 23%) Serious adverse events in 24.6% in remdesivir and 31.6% in placebo group
Goldman et al. [67]	Randomised, open-label, controlled trial (n = 397)	1. 5 days of remdesivir, 200 mg on the first day, followed by 100 mg once daily for 4 days (n = 200) 2. 10 days of remdesivir, 200 mg on the first day, followed by 100 mg once daily for 9 days (n = 197)	Primary: Clinical status on day 14 Secondary: Proportion of adverse events occurring on or after the first dose for up to 30 days after the last dose	Clinical improvement after 14 days in 65% of the 5-day course vs 54% of the 10-day course group Similar results in time to clinical improvement, recovery, and death between the groups after adjustment of baseline differences Mortality by day 14 in patients receiving mechanical ventilation or ECMO at day 5 was 40% in the 5-day group vs 17% in the 10-day group Adverse events in 70% of 5-day vs 74% in 10-day group
Spinner et al. [68]	Randomised, open-label, controlled trial (n = 584)	1. 10-day course of remdesivir, 200 mg on first day, followed by 100 mg daily (n = 197) 2. 5-day course of remdesivir, 200 mg on first day, followed by 100 mg daily (n = 199) 3. Standard of care (n = 200)	Primary: Distribution of clinical status on day 11 Secondary: Proportion of patients with adverse events throughout the time of the study	Higher odds of better clinical status distribution on day 11 in 5-day group in comparison to standard of care No statistically significant difference in clinical status distribution between 10-day and standard of care group Adverse events were 51% in the 5-day, 59% in the 10-day, and 47% in the standard of care group
WHO Solidarity Trial [69]	Randomised, open-label, controlled trial (n = 11,330), including treatment with remdesivir, hydroxychloroquine, lopinavir, interferon vs pairwise control	1. Remdesivir 200 mg on the first day and 100 mg on the following nice days (n = 2743) 2. Standard of care (n = 2708)	Primary: Effects on in-hospital mortality Secondary: Initiation of mechanical ventilation; duration of hospitalization	In-hospital mortality was 12.5% with remdesivir vs 12.7% receiving standard of care No reduction of initiation of ventilation among patients not already on ventilation with remdesivir

In the final report of the ACTT-1 trial, a randomised, double-blinded, placebo-controlled trial analysing remdesivir in hospitalised COVID-19 patients, participants from trial sites all over the world were enrolled to receive either remdesivir or a placebo. From 1114 patients screened for eligibility, 541 participants were allocated to receive remdesivir in the same dosage as the beforementioned study for 10 days. The placebo group consisted of 521 participants and received the same volume of placebo for 10 days. The median time from onset of symptoms to randomisation was 9 days. 90.1% of the patients enrolled had severe COVID-19, and the baseline characteristics did not differ between the groups. This study presented that the treatment with remdesivir expressed superiority in the time of recovery with a median time of 10 days in comparison to 15 days in the placebo group. This benefit of remdesivir was specifically seen in patients receiving baseline low-flow oxygen therapy. The mortality by day 15 was lower in the remdesivir group with 6.7% in comparison to 11.9% in the placebo group. The occurrence of serious adverse events was 24.6% in the remdesivir group, and 31.6% in the placebo group. These serious adverse events included respiratory failure occurring in 47 (8.8%) and 80 (15.5%) cases in the remdesivir and placebo group, respectively. The study concluded that remdesivir administered for 10 days had a benefit in hospitalised patients with COVID-19, especially in patients receiving low-flow oxygen at baseline [66] (Table 3).

To evaluate the efficacy of different treatment durations of intravenous remdesivir in COVID-19 patients hospitalised in 55 hospitals all over the world, a randomised, open-label clinical trial assigned 200 patients to receive remdesivir 200 mg on the first day and 100 mg per day on the following days for a total amount of 5 days, and 197 patients to receive the same dosing for a total of 10 days. Eligible participants were those with PCR-confirmed SARS-CoV-2 infection not beyond 4 days before randomisation, imaging-confirmed pneumonia, and patients with a SpO2 of not more than 94% or receiving supplemental oxygen. Patients with more severe baseline status were allocated to the longer-course group. After 14 days, a clinical improvement, defined as a change of two points on a seven-point ordinal scale, was seen in 65% of the 5-day course versus in 54% of the 10-day course group. After the baseline differences were adjusted, the two groups appeared to have similar results in time to clinical improvement, recovery, and death. The study did not conclude a significant difference of potency of the two administration courses in severe COVID-19 patients who did not require mechanical ventilation. But of the patients that developed a disease course requiring mechanical ventilation, a 10-day course of remdesivir was more beneficial [67] (Table 3). However, the complete efficacy of remdesivir cannot be ascertained in this study due to the lack of a control group.

The previous clinical trials concentrated on the efficacy of remdesivir in severe COVID-19 patients. But a randomised, open-label trial was concerned with the efficacy of remdesivir in moderately diseased COVID-19 patients. Eligible for this trial were hospitalised patients with PCR-confirmed SARS-CoV-2 infection, with pulmonary infiltrates and a SpO2 above 94% on room air. Remdesivir was dosed intravenously 200 mg on the first day and 100 mg once per day for following days. Of 596 patients randomised, 197 participants were grouped to receive remdesivir for 10 days, 199 for 5 days, and 200 patients were allocated to the standard care group. After 11 days, the odds of better clinical status were higher in the 5-day group compared to the standard care group. These findings were statistically significant, but the study concluded uncertainty if this difference could be clinically important. For the 10-day group there was no significant difference to the standard care group in clinical status distribution after 11 days. Furthermore, adverse events, such as headache, nausea, and hypokalaemia were more present in patients receiving remdesivir. The difference in the occurrence of adverse events, was only statistically significant between the 10-day and standard care group, but not between the 5-day and the standard care group. Serious adverse events were more commonly found in the standard care group with a larger difference between the standard care and 5-day course group, than between standard care and 10-day course group. The occurrence of death after 28 days was equally distributed between the groups and had no linkage to the treatment with remdesivir [68] (Table 3). This study provided information on the efficacy of remdesivir given for 5 days on moderately diseased COVID-19 patients, but the benefit is questionable.

In the recent report of the WHO solidarity trials from December 2020, remdesivir as well as the other tested repurposed antiviral agents, hydroxychloroquine, lopinavir and interferon beta-1a, did not reduce mortality, initiation of ventilation, nor hospital duration in patients hospitalised with COVID-19. In this open-label randomised clinical trial, in-hospital mortality of 2743 patients receiving remdesivir was 12.5% in comparison to 12.7% of 2708 patients receiving standard of care. 67% of patients in both the remdesivir and the control arm were receiving supplemental oxygen at trial entry. 24% of the remdesivir patients were receiving no supplemental oxygen at entry, whereas 25% in the control group. Participant characteristics of mechanical ventilation at entry was also equally distributed with 9% in each study group. Furthermore, there was no reduction of initiation of ventilation among patients not already on ventilation with remdesivir in comparison to standard of care [69] (Table 3).

The efficacy of remdesivir in the pharmacological treatment of COVID-19 remains questionable. Nevertheless, following the February 2021 update of the NIH COVID-19 Treatment Guidelines, remdesivir monotherapy is recommended with moderate rating to hospitalised COVID-19 patients requiring minimal supplemental oxygen. The combination of remdesivir with dexamethasone is recommended with moderate rating based on expert opinion to hospitalised patients requiring increased amounts of supplemental oxygen and who require oxygen delivery through high-flow devices or non-invasive ventilation. For hospitalised patients with COVID-19 who do not require supplemental oxygen the treatment with remdesivir is lacking data [70]. The ERS does not recommend remdesivir for hospitalised patients with COVID-19 requiring mechanical ventilation. Due to lack of reported benefits of randomised trials, the ERS is not making a recommendation for nor against the use of remdesivir in patients hospitalised with COVID-19 and not requiring invasive mechanical ventilation as of March 2021 [48].

## Pharmacological agents with immunomodulatory properties

### Tocilizumab

The cytokine storm plays an important role in the pathogenesis of severe COVID-19. A significant component of this cytokine storm, or cytokine release syndrome (CRS), is IL-6. Tocilizumab is a recombinant humanised monoclonal antibody that binds soluble and membrane-bound IL-6 receptors and inhibits its signal transduction. This pharmacological agent is used in the treatment of rheumatoid arthritis, as well as CRS and SIRS. These immunomodulatory features of tocilizumab are thought to alleviate the severity of symptoms in COVID-19 patients experiencing a CRS [71, 72].

In a retrospective, observational cohort of 544 patients with severe COVID-19 pneumonia performed in Italy, the administration of tocilizumab appeared to reduce the risk for invasive mechanical ventilation and death. 365 of these participants were receiving standard care alone, including the use of supplemental oxygen therapy, hydroxychloroquine, azithromycin, lopinavir/ritonavir or darunavir-cobicistat, and low-molecular weight heparin, if indicated by a physician. 179 participants received tocilizumab additionally to standard care. 88 of these 179 participants received intravenous tocilizumab 8 mg per kilogram of body weight with a maximum of 800 mg, twice, 12 h apart. 91 participants received tocilizumab subcutaneously, 162 mg in two doses that were administered at the same time once. As this study was not randomised, patients with more severe baseline characteristics were allocated to receive tocilizumab. After adjustment of these characteristics and the comparison between the groups, tocilizumab, in both administration routes, correlated with reduced risk of mechanical ventilation and death. On the other hand, tocilizumab use was also associated with a higher prevalence of new infections in comparison to standard care. This study declared a possible benefit of tocilizumab in severe COVID-19 patients and recommended a confirmation of these findings with randomised clinical trials [73] (Table 4).

**Table 4:** Summary of clinical studies: tocilizumab

Study	Study design (number of participants)	Study arms (number of participants)	Endpoints	Results
Guaraldi et al. [73]	Retrospective cohort study (n = 544)	1. Standard care plus tocilizumab intravenous 8 mg/kg (max. 800 mg) administered twice, 12 h apart, or subcutaneous 162 mg in two doses at once (n = 179) 2. Standard care (n = 365)	Primary: Composite of invasive mechanical ventilation or death	Initiation of invasive mechanical ventilation in 16% of standard care group vs 18% in the tocilizumab group Death occurred in 20% in standard care and 7% in tocilizumab group
Somers et al. [74]	Observational cohort study (n = 154)	1. Tocilizumab single dose 8 mg/kg (n = 78) 2. Control group (n = 76)	Primary: Survival probability after intubation Secondary: Clinical status at day 28	45% reduction in hazard of death and improved status on an ordinal outcome scale with tocilizumab Increased proportion of superinfections with tocilizumab (54% vs 26%)
Salama et al. [75]	Randomised, double-blinded, placebo-controlled trial (n = 377)	1. Tocilizumab 8 mg/kg in one or two doses (n = 249) 2. Placebo (n = 128)	Primary: Mechanical ventilation or death by day 28 Secondary: Time to hospital discharge or readiness for discharge; time to improvement of clinical status; time to clinical failure; death	Significantly lower percentage of patients who received mechanical ventilation or who died by day 28 with tocilizumab than with placebo (12% vs 19.3%) Similar median times in the tocilizumab group in comparison to placebo group in time to hospital discharge or readiness for discharge (6.0 days vs 7.5 days) and time to improvement in clinical status (6.0 days vs 7.0 days) Mortality by day 28 was 10.4% in the tocilizumab and 8.6% in the placebo group
REMAP-CAP Trial [76]	Randomised, open-label, controlled trial (n = 803)	1. Tocilizumab 8 mg/kg in one or two doses (n = 353) 2. Sarilumab 400 mg once (n = 48) 3. Standard of care (n = 402)	Primary: Number of respiratory and cardiovascular organ support–free days up to day 21 Secondary: 90-day survival; time to ICU and hospital discharge; improvement in the WHO ordinal scale at day 14	Median number of organ support–free days was 10 with tocilizumab, 11 with sarilumab, 0 with standard of care In-hospital mortality in the tocilizumab and sarilumab groups together was 27% in comparison to 36% in the control group Beneficial effects of tocilizumab and sarilumab in all secondary outcomes

Another observational cohort with 154 severe COVID-19 patients needing mechanical ventilation supports the use of tocilizumab as it correlated with improvement of survival. In comparison to a control group, the group receiving tocilizumab experienced a reduction of 45% in hazard of death. However, tocilizumab use was more frequently associated with superinfections, although, the case fatality rate between tocilizumab patients with or without superinfections was not significantly different [74] (Table 4).

In a randomised, doubled-blinded, placebo-controlled phase 3 trial, tocilizumab was evaluated for its safety and efficacy in COVID-19 patients. Eligibility criteria for enrolment included hospitalisation for COVID-19 pneumonia confirmed by PCR and radiographic imaging and SpO2 of less than 94% on ambient air breathing. Patients receiving continuous positive airway pressure, bilevel positive airway pressure, or mechanical ventilation were excluded from the trial. 249 patients were allocated to the tocilizumab group, receiving 8 mg per kilogram of body weight of tocilizumab in one or two doses intravenously, in addition to standard care according to local guidelines which could include antiviral treatment, systemic glucocorticoids, and supportive care. 128 patients were grouped to receive placebo in addition to standard care. Characteristics of the demographics and the disease of the patients at baseline were equally distributed among the two groups. In the assessment of the primary outcome of mechanical ventilation or death by day 28, the use of tocilizumab expressed a significant benefit in comparison to placebo. 12.0% of the tocilizumab group and 19.3% of the placebo group required mechanical ventilation or had died by day 28. Secondary outcomes of median times to hospital discharge or readiness for discharge, to improvement of clinical status, and to clinical failure were similar among the groups. Mortality from any cause by day 28 was 10.4% in the tocilizumab and 8.6% in the placebo group. The study suggests a benefit of tocilizumab in hospitalised patients with COVID-19 pneumonia with hypoxia and not receiving mechanical ventilation at treatment start and, possibly, an additional beneficial effect of tocilizumab to antiviral or corticosteroid therapy. In this trial, tocilizumab could reduce the likelihood of disease progression to the need of mechanical ventilation or death, but it did not improve survival [75] (Table 4).

In a preliminary report of the open-label randomised trial REMAP-CAP, the use of tocilizumab and sarilumab, both IL-6 receptor antagonists, in critically ill COVID-19 patients receiving respiratory or cardiovascular organ support was superior to standard of care. In this study, 353 patients were designated to the tocilizumab group, receiving the drug in a dosage of 8 mg per kilogram of body weight as an intravenous infusion over 1 h with a possible additional dosage 12 to 24 h later. 48 patients were allocated to receive sarilumab in a dosage of 400 mg, administered once as an intravenous infusion. 402 patients were assorted to the control group receiving standard care including corticosteroid therapy in more than 80% of the patients. Randomisation of COVID-19 patients occurred within 24 h of organ support in intensive care units and baseline characteristics of the patients were equally distributed across the groups. The results of the primary outcome of organ support-free days up to day 21 were 10 in the tocilizumab group, 11 in the sarilumab, and 0 in the control group. The use of tocilizumab and sarilumab was also associated with shorter time to clinical improvement and reduced in-hospital mortality in comparison to the control [76] (Table 4).

From the March 2021 statement of the NIH COVID-19 Treatment Guidelines, the use of tocilizumab in combination with dexamethasone is recommended in hospitalised patients with COVID-19 and rapid respiratory decompensation. This includes patients admitted to the ICU within 24 h and requiring invasive mechanical ventilation, non-invasive ventilation, and oxygen through high-flow nasal canula, and hospitalised non-ICU patients whose oxygen need is rapidly increasing requiring non-invasive ventilation or oxygen through high-flow nasal canula [77]. The ERS suggest the use of IL-6 receptor antagonist monoclonal antibodies in COVID-19 patients in the need of oxygen or ventilatory support. It is noted that the therapy with corticosteroids should have already been completed or initiated in these patients if not contraindicated. The ERS guidelines also note benefits of treatment start within 24 h of ventilatory support and in patients with high risk to progress to ventilatory support despite corticosteroid therapy [48].

### Corticosteroids

Another immunomodulatory option for the potential treatment of severe COVID-19 is the administration of corticosteroids. Although the use of corticosteroids in viral infection is questionable, their anti-inflammatory and antifibrotic actions could have beneficial effects on pneumonia, ARDS, and septic shock [78]. Previous studies evaluating corticosteroids in ARDS and septic shock patients concluded that corticosteroids could reduce mortality and duration of mechanical ventilation [78–81]. These insights give hope in the treatment of severe and mechanically ventilated COVID-19 patients with corticosteroids to wean the patients off from respiratory support and to reduce mortality.

In a report of the results of a controlled, open-label trial that is part of the RECOVERY trial in the United Kingdom, 6425 eligible patients were randomised to receive either usual care or usual care with additional dexamethasone. 2104 patients were allocated to receive dexamethasone, 6 mg once per day for 10 days or until hospital discharge administered orally or intravenously. 4321 patients were grouped to receive usual care. Patients that were eligible for this trial were those with clinically suspected or laboratory confirmed SARS-CoV-2 infection and had no contraindications for the use of corticosteroids. After a primary outcome assessment of 28-day mortality, the group receiving mechanical ventilation or just supplemental oxygen therapy without mechanical ventilation at the time of randomisation in the dexamethasone group had a lower mortality rate than those in the usual care group. For patients that did not need respiratory support at the time of randomisation, the use of dexamethasone was not significantly beneficial in comparison to usual care. Furthermore, the reduction of mortality was experienced more in individuals that had symptoms for more than 7 days but not in those with recent symptom onset which possibly correlates to the prevalence of inflammatory lung damage. In this study, the use of dexamethasone was associated with lower risk of patients receiving oxygen to deteriorate to invasive mechanical ventilation and a greater likelihood of patients already on invasive mechanical ventilation to successful weaning [82] (Table 5). The RECOVERY trial proved a benefit of low-dose dexamethasone in mechanically ventilated and oxygen-needing, not mechanically ventilated COVID-19 patients.

**Table 5:** Summary of clinical studies: corticosteroids

Study	Study design (number of participants)	Study arms (number of participants)	Endpoints	Results
Recovery Trial [82]	Randomised, open-label, controlled trial (n = 6425)	1. Dexamethasone 6 mg once daily for 10 days (n = 2104) 2. Standard of care (n = 4321)	Primary: 28-day all-cause mortality Secondary: Time until hospital discharge; receipt of invasive mechanical ventilation; death	Significantly lower 28-day mortality in the dexamethasone group than in standard of care (22.9% vs 25.7%). Greater benefit in patients receiving invasive mechanical ventilation at baseline Shorter duration of hospitalization in the dexamethasone group (median, 12 days vs 13 days) Lower progression in the dexamethasone group to invasive mechanical ventilation in patients not receiving invasive mechanical ventilation at baseline
Tomazini et al. CoDEX trial [83]	Randomised, open-label, controlled trial (n = 299)	1. Dexamethasone 20 mg daily for 5 days, followed by 10 mg daily for 5 days (n = 151) 2. Standard of care (n = 148)	Primary: Ventilator-free days during the first 28 days Secondary: All-cause mortality at 28 days, 15-day clinical status; ICU-free days during the first 28 days, mechanical ventilation duration at 28 days; SOFA scores at 48 h, 72 h, and 7 days	Significantly higher mean number of days alive and free from mechanical ventilation in the dexamethasone group (6.6 days vs 4.0 days) No statistically significant difference in all-cause 28-day mortality (56.3% in dexamethasone vs 61.5% in control), ICU-free days at day 18 (mean, 2.1 in dexamethasone vs 2.0 in control), and duration of mechanical ventilation (12.5 days in dexamethasone vs 13.9 days in control) Significantly lower SOFA score at day 7 in the dexamethasone group
Dequin et al. [84]	Randomised, double-blind, placebo-controlled trial (n = 149)	1. Low-dose hydrocortisone 200 mg daily for 7 days, then 100 mg daily for 4 days and 50 mg daily for 3 days (n = 76) 2. Placebo (n = 73)	Primary: Treatment failure at day 21 (death or persistent dependency on mechanical ventilation or high-flow oxygen therapy) Secondary: Use of tracheal intubation; use of prone position, ECMO or inhaled nitric oxide; PaO2:FIO2 ratio at days 1 to 7 and on days 14 and 21; proportion of patients with and number of episodes of nosocomial infections	No statistically significant benefit of low-dose hydrocortisone in critically ill COVID-19 patients with acute respiratory failure Treatment failure at day 21 was 42.1% in the hydrocortisone group vs 50.7% in the placebo group) Requirement of intubation was 50% in the hydrocortisone and 75% in the placebo group No significant difference in use of prone positioning between the groups (47.4% in hydrocortisone vs 53.4% in placebo group) No significant difference in PaO2:FIO2 ratio trend between the groups Occurrence of at least one nosocomial infection by day 28 was 37.3% in the hydrocortisone vs 41.1% in the placebo group
REMAP-CAP trial [85]	Randomised, open-label, controlled trial (n = 384)	1. Hydrocortisone 50 or 100 mg every 6 h for 7 days (n = 137) 2. Shock-dependent course of hydrocortisone 50 mg every 6 h (n = 146) 3. No hydrocortisone (n = 101)	Primary: Organ support–free days within 21 days Secondary: In-hospital mortality; ICU and hospital length of stay; respiratory support–free days, cardiovascular organ support–free days; progression to invasive mechanical ventilation, ECMO or death	Median organ-support free days was 0 in fixed-dose hydrocortisone, 0 in shock-dependent hydrocortisone, and 0 in the control group The probabilities of superiority were 93% and 80% in the fixed-dose hydrocortisone and in the shock-dependant hydrocortisone groups, respectively, in comparison to the no-hydrocortisone group In-hospital mortality was 30% in fixed-dose, 26% in shock-dependant, and 33% in no-hydrocortisone group
Jeronimo et al. Metcovid [86]	Randomised, double-blind, phase IIb, placebo-controlled trial (n = 393)	1. Methylprednisolone 0.5 mg/kg twice daily for 5 days (n = 194) 2. Placebo (n = 199)	Primary: 28-day mortality Secondary: Early mortality at days 7 and 14; need for intubation by day 7; proportion of patients with PaO2/FiO2 < 100 by day 7	No significant difference in primary or secondary outcomes between the groups 28-day mortality was 37.1% in the methylprednisolone vs 38.2% in the placebo group

The CoDEX multicentre, randomised, open-label clinical trial in Brazil supports the findings of the RECOVERY trial. This trial enrolled 299 ICU patients with confirmed or suspected COVID-19 with moderate to severe ARDS. Randomisation allocated 148 patients to receive standard care and 151 patients to receive standard care with dexamethasone, intravenously 20 mg per day for 5 days, followed by 10 mg per day for another 5 days or, if occurring earlier, until discharge from the intensive care unit. After a follow-up of 28 days, the dexamethasone group had a statistically significant longer time of days being free from mechanical ventilation and being alive with a mean time of 6.6 days in comparison to 4.0 days in the standard care group. Also, the dexamethasone group had a significantly lower mean Sequential Organ Failure Assessment (SOFA) score than the comparison group. Secondary outcome assessments, such as mortality of all causes, ICU-free days, and duration of mechanical ventilation after 28 days, as well as clinical status on a six-point ordinal scale at day 15, had no statistically significant difference between the groups. Furthermore, the occurrence of secondary infections, adverse events, and insulin need for glucose control was similar between the groups [83] (Table 5). This trial was terminated early due to the findings of the previously mentioned trial and, thus, did not reach their goal sample size, which could explain the lack of positive secondary outcome confirmation. Nevertheless, the CoDEX trial provided evidence on the benefit of high-dose dexamethasone in COVID-19 patients with moderate to severe ARDS.

In a multicentre, randomised, double-blinded clinical trial in France, the treatment efficacy of low-dose hydrocortisone was evaluated in critically ill ICU patients with acute respiratory failure due to COVID-19. 76 patients were assigned to receive hydrocortisone, administered intravenously, 200 mg per day for 7 days, followed by 100 mg per day for the next 4 days, and then 50 mg per day for another 3 days. 73 participants received a placebo of saline solution. The primary outcome assessed was the treatment failure at day 21, which was determined as the occurrence of death, continued mechanical ventilation, or persistence of high-flow oxygen therapy. The hydrocortisone group had comparatively less treatment failure events with 42.1%, versus 50.7% in the placebo group. However, this difference was marked as not statistically significant. Also, there was no statistically significant difference in the evaluation of secondary outcomes, including the need of tracheal intubation in non-intubated patients at time of randomisation, the need of prone positioning, ECMO, or nitric oxide inhalation, the ratio of arterial oxygen partial pressure to fractional inspired oxygen (PaO_2_:FIO_2_) at days 1 to 7 and on days 14 and 21, and the post hoc analysis of cumulative proportions of nosocomial infections for up to day 28. This study concluded no statistically significant benefit of low-dose hydrocortisone in critically ill COVID-19 patients with acute respiratory failure [84] (Table 5). Although this trial had good potential in delivering significant information due to its placebo-controlled layout, it was also terminated early because of the findings of the RECOVERY trial and had therefore not reached their target sample size which could have affected the results of the study. Similar results were presented by the REMAP-CAP trial on low-dose hydrocortisone in intensive care COVID-19 patients, but the study was also terminated early [85] (Table 5).

In the Brazilian randomised, double-blinded, phase IIb, placebo-controlled trial, named Metcovid, the efficacy of a short course of high-dose methylprednisolone was investigated in patients hospitalised for suspected COVID-19. Patients that were enrolled were adults that had a SpO2 of 94% or less, those receiving supplementary oxygen therapy, or those receiving invasive mechanical ventilation and had a suspected or confirmed SARS-CoV-2 infection. RT-PCR confirmation of SARS-CoV-2 infection was presented in 81.3% of the patients. 194 participants received 0.5 mg per kilogram of intravenous methylprednisolone two times daily for 5 days, whereas 199 patients received a placebo of saline solution. The median time from disease onset to randomisation was 13 days in both groups. The assessment of the primary outcome of mortality at day 28 demonstrated equal results between the groups, although patients above the age of 60 in the methylprednisolone group appeared to have lower mortality and benefitted from corticosteroid use. The patients in this age group had generally higher C-reactive protein levels and more distinct systemic inflammation [86] (Table 5). In comparison to the results of the RECOVERY trial, this study could not declare a definite benefit of corticosteroids in the treatment of the enrolled patients. However, the treatment courses were different between these trials and patients in the Metcovid trial were treated with corticosteroids only for 5 days in comparison to 10 days in the RECOVERY trial.

From the February 2021 update of the COVID-19 Treatment Guidelines, the Panel strongly recommends dexamethasone for hospitalised COVID-19 patients who require supplemental oxygen through high-flow devices or non-invasive ventilation. A combination with remdesivir is also mentioned as a treatment option for these patients. Dexamethasone is also strongly recommended for hospitalised patients on invasive ventilation or ECMO. For hospitalised patients who require increasing amounts of supplemental oxygen not through a high-flow device, non-invasive ventilation, invasive mechanical ventilation, or ECMO the use of dexamethasone can optionally be added to the treatment of remdesivir or can be administered as monotherapy if a combination with remdesivir is not feasible. Furthermore, the Panel strongly recommends against the use of dexamethasone for hospitalised COVID-19 patients who do not need supplemental oxygen and to non-hospitalised, mild to moderate COVID-19 patients. Recommended alternatives for dexamethasone, if not available, are prednisone, methylprednisolone, and hydrocortisone [70]. The ERS supports this viewpoint by strongly suggesting systemic corticosteroids to patients who are hospitalised for COVID-19 that need oxygen, non-invasive ventilation, or invasive mechanical ventilation [48].

## Adjunctive treatment strategies

### Anticoagulation

COVID-19 has potential in causing severe inflammation leading to cytokine storm, SIRS, and multiple organ failure. These factors, as well as the disrupted alveolar-endothelial barrier, possible infection of endothelial cells by the virus, and additional factors, such as immobilisation and comorbidities of patients, give rise to an increased risk of thromboembolic complications [21–23].

ARDS is an important component of the disease pathogenesis of COVID-19. A previous prospective pilot study, published in 2016, already linked ARDS with coagulopathy [87]. Mechanically ventilated ARDS patients had significantly higher median plasma concentrations of tissue factor and plasminogen activator inhibitor-1 at day 7 of ICU treatment in comparison to non-ARDS patients. Pathological investigations of deceased COVID-19 patients support the theory of hypercoagulation by the findings of increased incidences of pulmonary microthrombi [88]. Furthermore, in pulmonary autopsies of ARDS patients who were infected with COVID-19 in comparison to influenza H1N1-associated ARDS patients, lungs from both groups presented with diffuse alveolar damage and infiltrating perivascular lymphocytes, but the COVID-19 affected lungs had more severe endothelial injury with intracellular virus, disrupted endothelial cell membranes, widespread vascular thrombosis with microangiopathy, occlusion of alveolar capillaries, and significantly more new vessels through intussusceptive angiogenesis [89].

A retrospective cohort study in Wuhan, China demonstrated that in 183 patients admitted to the hospital between January to February 2020 with confirmed COVID-19 pneumonia, non-survivors presented significantly higher D-dimer and fibrin degradation products, and longer prothrombin time at the time of admission in comparison to survivors. These factors correlated significantly with disease prognosis. 71.4% of the non-survivors also met criteria for overt disseminated intravascular coagulation (DIC) according to the International Society on Thrombosis and Haemostasis diagnostic criteria [90]. The association of these abnormal coagulation markers with disease severity, ICU-admission, and mortality in COVID-19 patients has been supported by several cohort studies in China and in the United States [16, 91–93].

In a Chinese retrospective cohort analysis of 449 severe COVID-19 patient, 99 patients received concurrent heparin treatment for 7 days. 94 of these 99 patients received low molecular weight heparin (LMWH). Although, the 28-day mortality was not different between patients receiving heparin and those who did not, a benefit was experienced in patients with sepsis-induced coagulopathy score of four and more, and in patients with markedly increased D-dimers of more than six-fold of the upper limit of normal. These patients had a significantly lower 28-day mortality when heparin was used [94].

An Irish prospective cohort study suggests that the coagulopathy occurring in COVID-19 in early stages is rather distinct from typical DIC and that the diffuse pulmonary bilateral inflammation in COVID-19 leads to a pulmonary-specific vasculopathy, termed pulmonary intravascular coagulopathy (PIC). In the mainly Caucasian population of this cohort, a prophylactic dose of LMWH was administered concurrently to the study and no systemic DIC was reported. The study additionally proposes that the administration of prophylactic dose LMWH does not have a significant impact on increasing D-dimer levels and that further randomised controlled studies should evaluate more intensive dosing strategies in severe COVID-19 patients with PIC presentation [95]. This suggestion is advocated by a French prospective cohort in which 150 patients with COVID-19 ARDS were assessed for thrombotic risk in comparison to non-COVID-19 ARDS patients. None of the 150 COVID-19 patients developed overt DIC, but despite the administration of prophylactic or therapeutic anticoagulation, thromboembolic complications were highly prevalent and significantly more than in non-COVID-19 ARDS patients. The most common thromboembolic event was pulmonary embolism, occurring in 16.7% of the 150 COVID-19 patients [96]. These findings suggest that close monitoring of severe COVID-19 patients with ARDS for coagulation markers and treatment of developing PIC with higher dose anticoagulation should be evaluated in randomised clinical trials for the potential decrease of disease mortality.

As of March 2021, The NIH cannot provide verified recommendations for anticoagulative therapy strategies in COVID-19 patients due to the lack of data from randomised trials. Nevertheless, based on expert opinion, the Panel strongly recommends venous thromboembolism prophylaxis for hospitalised COVID-19 patients as per the standard of care for other hospitalised patients. At the time of this review, the use of thrombolytics or increased doses for venous thromboembolism prophylaxis in COVID-19 patients outside of clinical trials is neither recommended for nor against. The treatment of COVID-19 patients with thromboembolic events should be commenced with therapeutic anticoagulative therapy as per the standard of care for non-COVID-19 patients. Those COVID-19 patients requiring ECMO or continuous renal replacement therapy, and those with catheter or extracorporeal filter thrombosis should be treated with antithrombotic therapy as per standard institutional protocol for non-COVID-19 patients [97]. The ERS supports the recommendation of offering a form of anticoagulation to hospitalised COVID-19 patients but are not able to make a recommendation regarding dose or type of anticoagulative therapy due to lack of data as of March 2021 [48].

## Discussion

The hurry in finding a cure for COVID-19 has brought problems with the acceptance of some clinical trials due to arguments about methodological incorrectness. The lack of a clear consensus on primary and secondary outcomes and endpoints, as well as inclusion criteria for enrolment of clinical trials testing for pharmacological treatment of COVID-19 make it difficult to interpret and compare the results. Furthermore, whereas clinical trials in randomised, blinded, placebo-controlled fashion provide the little biased information of novel treatment strategies, these trial goals were not always feasible during the pandemic. What resulted in was a mixture of different approaches to find pharmacological agents for COVID-19 treatment that were not always presented without their limitations.

In total, 97 references were studied and used to conduct this research paper. Due to the vast number of published articles on this new topic, not all research papers found in the utilised databases could be taken into consideration. Articles prioritised for selection were those that were most recently published and peer reviewed by the time of the conduction of this literature review. Research papers providing information on COVID-19 pharmacological treatment that had not been peer reviewed, yet, had to be excluded.

In vitro analyses of SARS-CoV-2, as well as previous studies of SARS and MERS treatment became guides for the direction of possible COVID-19 treatment. Taken by the example of the in vitro analyses of chloroquine and hydroxychloroquine, it appears to be indisputable that investigations in SARS-CoV-2-infected human airway epithelial cells should have led the discussion before the proposal of clinical trials and that human airway epithelial cells for SARS-CoV-2 in vitro drug analyses should become standard procedure [45, 46].

The results of clinical trials surrounded with the topic of drug treatment of COVID-19 with chloroquine or hydroxychloroquine with or without azithromycin precipitated the most controversy. Whereas a handful of clinical trials advocated the use of chloroquine in the treatment of COVID-19, other trials reported no proven benefit of chloroquine in COVID-19 patients [33–35]. Hydroxychloroquine for the treatment of mild to moderate COVID-19, as well as the observations of risk of intubation or death in SARS-CoV-2-positive, hospitalised patients did not conclude a benefit of hydroxychloroquine treatment in COVID-19 patients [36, 37]. The combination of hydroxychloroquine and azithromycin for the use of SARS-CoV-2 viral load reduction has been advocated by a French clinical trial lead by Gautret and colleagues [38]. However, the results of this study and the effect of the drug combination on patients’ viral load have been opposed by critics and negated by studies [39–41]. Furthermore, no effect of clinical improvement was ascribed to the use of hydroxychloroquine and azithromycin in mild to moderate COVID-19 patients [42]. As a possible postexposure prophylaxis, hydroxychloroquine has not proven to have sufficient effect on a population of mainly health care workers [43]. Both chloroquine and hydroxychloroquine, especially in high dosing and in the combination with azithromycin, have potential in causing cardiac side effects and prolonged QTc, giving rise to a significant risk in causing life-threatening arrhythmia [33, 41, 42, 46]. Considering the given data, neither chloroquine, nor hydroxychloroquine with or without azithromycin present clear proven clinical benefit for the treatment of COVID-19.

The drug combination lopinavir/ritonavir has also not presented convincing efficacy in the treatment of COVID-19. While an early administration of lopinavir/ritonavir within 10 days of symptom onset, as observed in an observational cohort, could potentially decrease time of SARS-CoV-2 shedding, randomised controlled trials could not present efficacy in improving clinical status nor in shortening pharyngeal viral detectability in COVID-19 patients [52, 54, 55]. Moreover, a pharmacokinetic analysis of lopinavir/ritonavir in standard dosing concluded inefficient plasma concentrations for the inhibition of SARS-CoV-2 replication [57]. Given the evidence, lopinavir/ritonavir appears to be ineffective in treating SARS-CoV-2 infections.

Randomised controlled trials provide proof on the efficacy and safety of remdesivir in hospitalised COVID-19 patients requiring supplemental oxygen therapy [66–68]. Specifically, the ACTT-1 trial supports the use of remdesivir in COVID-19 patients receiving low-flow oxygen therapy as it expressed superiority in the time of recovery in comparison to the control [66]. On the other hand, the results of the WHO Solidarity trial did not present clinical benefits of remdesivir treatment reducing mortality, initiation of ventilation, nor hospital duration in patients hospitalised with COVID-19 [69]. At the time of the conduction of this research paper, the NIH recommends remdesivir monotherapy only in patients hospitalised with COVID-19 who require minimal supplemental oxygen [70]. In contrary, the ERS makes no recommendation on the treatment of hospitalised patients with COVID-19 who do not require invasive mechanical ventilation [48]. Results of further randomised trials are needed to support the results of the ACTT-1 trial. Also, more trials should be conducted on the efficacy and safety of remdesivir and dexamethasone combination therapy in COVID-19 patients. Remdesivir is the first and, as of the time of this review, the only antiviral agent that is recommended for the clinical treatment of COVID-19 patients. As the use of remdesivir is becoming of more clinical importance during the COVID-19 pandemic, clinical trials investigating possible side effects of the usage of remdesivir in COVID-19 patients, such as hypokalaemia, are of utter importance and should as well be proposed.

Drugs acting against the cytokine storm of COVID-19 have good potential in decreasing the mortality in theory. Following the recent results of randomised clinical trials, tocilizumab seems to be a promising agent in the pharmacological treatment of COVID-19 by reducing the likelihood of disease progression, time to clinical improvement, and mortality, specifically in patients admitted to ICU within 24 h on organ support [75, 76]. The NIH as well as the ERS have spoken out about a treatment recommendation of COVID-19 including tocilizumab [48, 77]. Future research should focus on the benefits of a possible drug combination with dexamethasone in severe COVID-19 patients and the optimal timing and target group of tocilizumab initiation.

The RECOVERY trial has delivered positive results on the clinical efficacy of low-dose dexamethasone in mechanically ventilated and non-mechanically ventilated, but supplemental oxygen requiring COVID-19 patients [82]. Systemic corticosteroids, specifically dexamethasone, are the treatment modality with the most evidence on the efficacy against severe COVID-19 and are included in the treatment guidelines of the NIH and ERS [48, 70].

Thromboembolic events are serious complications of severe COVID-19 cases [20]. As of March 2021, thromboprophylaxis and thromboembolic treatment are recommended for COVID-19 patients as per standard protocol for regular ward or intensive care patients [97]. Further research in the areas of the COVID-19-specific coagulopathy and testing of higher dose anticoagulation, possibly in combination with immunotherapy, in randomised controlled trials is necessary to gain data about possible improvements of mortality rates in severe COVID-19 cases.

Furthermore, the spread of SARS-CoV-2 and the search of its origin has put attention to the topicality of zoonotic transmissions of infectious diseases. By spending more research on the exact origin of SARS-CoV-2 and the prevention of zoonotic transmissions, future epidemic- or pandemic-causing infectious diseases could potentially be prevented, possibly saving future lives [9].

## Conclusions

In conclusion, the most beneficial pharmacological agent for the treatment of COVID-19 are corticosteroids, especially dexamethasone, for the treatment of mechanically ventilated COVID-19 patients. Other promising agents are remdesivir for the treatment of patients with COVID-19 pneumonia requiring minimal supplemental oxygen therapy, and IL-6 receptor antagonist monoclonal antibodies in severe COVID-19. Lopinavir/ritonavir, as well as chloroquine or hydroxychloroquine with or without azithromycin demonstrate the least efficacy in the treatment of COVID-19. The clinical benefits of the treatment of a COVID-19-specific coagulopathy with increased dosing of anticoagulation need further research and confirmation of randomised controlled trials.

The COVID-19 pandemic, as devastating as it is, is teaching humanity an important lesson in dealing with crises. Instead of getting defeated by this pandemic, we need to evaluate what we have done correctly and admit what we have done wrong to be prepared for the next crisis.

## Data Availability

Not applicable.

## Abbreviations

3CLpro: 3-Chymotrypsin-like protease
ACE2: Angiotensin-converting enzyme two
ARDS: Acute respiratory distress syndrome
CoV, CoVs: Coronavirus, Coronaviruses
COVID-19: Coronavirus disease 2019
CRS: Cytokine release syndrome
CT: Computed tomography
DIC: Disseminated intravascular coagulopathy
E: Envelope protein
ECMO: Extracorporeal membrane oxygenation
ERS: European Respiratory Society
ICU: Intensive care unit
IL: Interleukin
LMWH: Low molecular weight heparin
M: Membrane protein
MERS: Middle East respiratory syndrome
N: Nucleocapsid protein
NIH: National Institutes of Health
PaO_2_:FIO_2_: Arterial oxygen partial pressure to fractional inspired oxygen
PCR: Polymerase chain reaction
PIC: Pulmonary intravascular coagulopathy
RT-PCR: Reverse transcription polymerase chain reaction
PLpro: Papain-like protease
QTc: Corrected QT interval
RdRp: RNA-dependent RNA polymerase
RNA: Ribonucleic acid
S: Spike protein
SARS: Severe acute respiratory syndrome
SARS-CoV-2: Severe acute respiratory syndrome coronavirus two
SIRS: Systemic inflammatory response syndrome
SOFA: Sequential Organ Failure Assessment
SpO2: Peripheral capillary oxygen saturation
TMPRSS2: Transmembrane protease serine subtype two
WHO: World Health Organization
